# Genome-Wide Identification and Expression Profiling of Heavy Metal ATPase (HMA) Genes in Peanut: Potential Roles in Heavy Metal Transport

**DOI:** 10.3390/ijms25010613

**Published:** 2024-01-03

**Authors:** Jinxiu Li, Zheng Zhang, Gangrong Shi

**Affiliations:** College of Life Sciences, Huaibei Normal University, Huaibei 235000, China; 12111070720@chnu.edu.cn (J.L.); swx@chnu.edu.cn (Z.Z.)

**Keywords:** *Arachis hypogaea*, P_1B_-ATPase, metal translocation, genome-wide identification, gene expression

## Abstract

The heavy metal ATPase (HMA) family belongs to the P-type ATPase superfamily and plays an essential role in the regulation of metal homeostasis in plants. However, the gene family has not been fully investigated in peanut. Here, a genome-wide identification and bioinformatics analysis was performed on *AhHMA* genes in peanut, and the expression of 12 *AhHMA* genes in response to Cu, Zn, and Cd was evaluated in two peanut cultivars (Silihong and Fenghua 1) differing in Cd accumulation. A total of 21 *AhHMA* genes were identified in the peanut genome, including ten paralogous gene pairs derived from whole-genome duplication, and an additional gene resulting from tandem duplication. AhHMA proteins could be divided into six groups (I–VI), belonging to two clades (Zn/Co/Cd/Pb-ATPases and Cu/Ag-ATPases). Most AhHMA proteins within the same clade or group generally have a similar structure. However, significant divergence exists in the exon/intron organization even between duplicated gene pairs. RNA-seq data showed that most *AhHMA* genes are preferentially expressed in roots, shoots, and reproductive tissues. qRT-PCR results revealed that *AhHMA1.1*/*1.2*, *AhHMA3.1*/*3.2*, *AhHMA7.1*/*7.4*, and *AhHMA8.1* might be involved in Zn transport in peanut plants, while *AhHMA3.2* and *AhHMA7.5* might be involved in Cd transport. Our findings provide clues to further characterize the functions of *AhHMA* genes in metal uptake and translocation in peanut plants.

## 1. Introduction

Heavy metal (HM) pollution has become one of the major environmental concerns in the world. Some HMs, such as zinc (Zn), copper (Cu), manganese (Mn), cobalt (Co), and nickel (Ni), are essential for plants, serving structural roles in proteins, acting as enzyme cofactors and playing a part in cellular redox reactions. Other HMs, such as cadmium (Cd) and lead (Pb), are non-essential elements that are highly toxic to plants. The deficiency of essential metals negatively affects plant growth and development. However, when excessively accumulated, they can cause toxicity [1]. Thus, plants have to establish a series of regulatory mechanisms to precisely maintain the homeostasis of essential metals. Generally, HM accumulation in plant tissues is controlled by the following processes: (i) the symplastic uptake by roots; (ii) radial transport; (iii) xylem loading; (iv) root-to-shoot translocation; and (v) intracellular mobilization, trafficking, and sequestration [2,3,4]. Most of these processes are mediated by numerous membrane transport proteins belonging to different protein families, including heavy metal ATPases (HMAs).

HMAs, also named P_1B_-ATPase, belong to the P-type ATPase superfamily (E1–E2 ATPases) that shares a common enzymatic mechanism to transport diverse sets of ions across membranes using the energy generated from ATP hydrolysis. HMAs structurally differ from other P-type ATPases, containing six–eight transmembrane helices and characterized by a CPx/SPC motif in the sixth transmembrane domain (TMD) and putative metal-binding domains (MBDs) at the N- and C-termini [5,6]. Phylogenetically, HMAs can be classified into six clusters (1–6) that are responsible for transporting Cu/Ag (clusters 3–6) or Zn/Cd/Co/Pb (clusters 1–2) [5,7].

Since the first plant *HMA* genes were cloned from *Arabidopsis* (*AtHMA6*/*PAA1*) [8], multiple *HMA* genes were identified in several plant species such as *Arabidopsis* and rice (*Oryza sativa*) [5,7]. In *Arabidopsis*, AtHMA1 and AtHMA6/PAA1 localize to the chloroplast envelope and behave as distinct pathways to import copper in the chloroplast [9,10]. AtHMA8/PAA2 localizes at the thylakoid membranes and is responsible for transporting Cu into the thylakoid lumen [11]. AtHMA1 is also shown to be involved in Zn detoxification by reducing the Zn content in the chloroplast [12]. AtHMA5 contributes to Cu detoxification in roots and/or xylem loading for root-to-shoot Cu translocation [13,14]. AtHMA7/RAN1 plays a crucial role in delivering Cu^+^ across post-Golgi membranes to increase ethylene-binding activity [15,16]. AtHMA2 and AtHMA4 are plasma membrane (PM)-localized transporters that are responsible for long-distance translocation of Cd and Zn by loading them to the xylem [17,18,19]. AtHMA3 is a vacuolar membrane-localized transporter participating in the detoxification of Cd, Zn, Co, and Pb by vacuolar sequestration [20].

Rice has nine *HMA* genes and most of them have been functionally characterized. OsHMA2 localizes in the PM and is involved in root-to-shoot translocation of Zn and Cd by mediating the xylem loading [21,22]. OsHMA3 is a tonoplast-localized transporter and plays a role in the sequestration of Cd and Zn into vacuoles in root cells [23,24]. OsHMA4 is also a tonoplast-localized transporter that functions to sequester Cu into root vacuoles [25]. OsHMA5 is localized to the PM and involved in loading Cu to the xylem for root-to-shoot translocation [26]. OsHMA9 is localized to the PM and considered to function for Cu, Zn, and Pd efflux from the cytoplasm [27]. OsHMA7 might be involved in Fe and Zn translocation in rice [28].

Peanut (*Arachis hypogaea* L., 2n = 4x = 40) is one of the economically and nutritionally major oilseed crops. It provides both edible oil and vegetable protein for people all over the world. Peanut is a Cd-tolerant species and can accumulate Cd even at low Cd levels, being an excellent candidate for phytoremediation [29]. Extensive studies have shown a significant variation in Cd accumulation and tolerance within peanut cultivars [2,29]. Besides, differential accumulation and translocation of Fe, Zn, and Mn were also observed between two peanut cultivars (Fenghua 1 and Silihong) [30,31]. Recently, several gene families of metal transporters such as zinc/iron-regulated transporter-like proteins (*ZIP*s), oligopeptide transporters (*OPT*s), natural resistance-associated macrophage proteins (*NRAMP*s), and metal tolerance proteins (*MTP*s), have been identified in the peanut genome [30,31,32,33]. However, little is known about the roles of the *HMA* family genes in metal accumulation in peanut. Identifying metal transporter genes is essential for screening or breeding high metal-accumulating cultivars for phytoremediation or low metal-accumulating cultivars for safe food production in heavy metal-contaminated soils.

Currently, the whole genome sequences of the cultivated peanut (cv. *Tifrunner*) and the two wild ancestral species (*A. duranensis* and *A. ipaënsis*) have been released [34,35]. This makes it possible to identify gene families at the whole genome level. Here, a systematic genome-wide analysis was performed to identify *AhHMA* family genes in peanut and to characterize their phylogeny, structure and function. Additionally, two peanut cultivars (Silihong and Fenghua 1) differing in Cd accumulation [36] were used for evaluating the expression of *AhHMA* genes in response to HMs (Cu, Zn, and Cd). The findings provide clues to functionally characterize *AhHMA* genes and shed some light on the molecular mechanisms of metal homeostasis in peanut.

## 2. Results

### 2.1. Identification of the AhHMA Gene Family in Peanut

The BLASTP against the peanut genome database using AtHMA and OsHMA protein sequences as queries resulted in 87 non-redundant protein sequences. After removing sequences without E1–E2 ATPase (PF00122) and hydrolase (PF00702) domains, 62 proteins remained. Phylogenetic analysis indicated that these proteins were classified into three clades (Figure S1). Among them, one clade includes 21 proteins that were clustered together with AtHMAs and OsHMAs, which can be considered as potential HMAs. Multiple sequence alignment indicated that all these HMAs have conserved HMA motifs, including TGE, DKTGT, CPC/SPC, and GDGxNDxP (Figure S2). Thus, we believe that the peanut genome contains 21 putative HMA genes, including two homologous genes of *AhHMA1* (*AhHMA1.1*/*1.2*), two homologous genes of *AhHMA3* (*AhHMA3.1*/*3.2*), eight homologous genes of *AhHMA5* (*AhHMA*s*5.1–5.8*), two homologous genes of *AhHMA6* (*AhHMA6.1*/*6.2*), five homologous genes of *AhHMA7* (*AhHMA*s*7.1–7.5*), and two homologous genes of *AhHMA8* (*AhHMA8.1*/*8.2*) (Table 1). The length of *AhHMA* genes varied from 4665 bp (*AhHMA5.1*) to 16,396 bp (*AhHMA5.7*), with CDS length from 2379 bp (*AhHMA8.2*) to 4041 bp (*AhHMA5.6*). The amino acid number of AhHMA proteins ranged from 792 (AhHMA8.2) to 1346 (AhHMA5.6), and the molecular weight of AhHMA proteins varied from 84.17 kDa (AhHMA8.2) to 149.65 kDa (AhHMA5.6). The instability index for all AhHMA proteins is less than 40 (30.82–39.05), suggesting a high stability in vitro. The aliphatic index ranged from 87.62 to 106.87, implying a high content of aliphatic amino acids and stability over a wide temperature range. The GRAVY for most of AhHMAs is higher than 0, indicating that AhHMAs are hydrophobic proteins. The pI values of most members less than 7 (Table 1). The number of TMDs varied from 6 to 9, and most AhHMAs contained 8 TMDs (Table 1). AhHMA1.1/1.2, AhHMA6.1/6.2, and AhHMA8.1/8.2 were predicted to localize to chloroplast, while the remaining members localize to plasma membrane (Table 1).

### 2.2. Phylogenetic Analysis of HMA Genes

**Figure 1:**
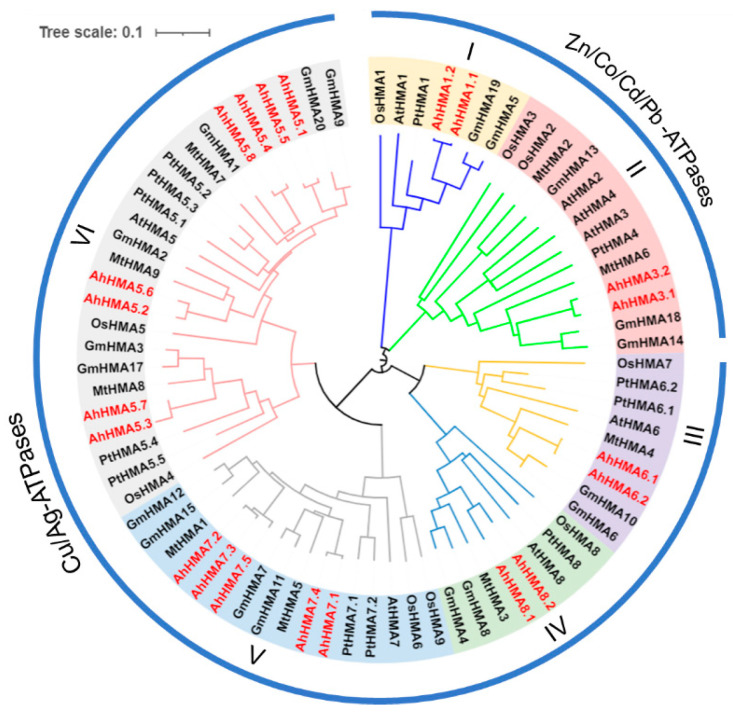
Phylogenetic relationships of HMA proteins in peanut and other plant species. Red color represents the 21 AhHMA proteins of peanut. The species involved in the evolutionary tree include *Arabidopsis thaliana* (AtHMAs), *Oryza sativa* (OsHMAs), *Glycine max* (GmHMAs), *Medicago truncatula* (MtHMAs), and *Populus trichocarpa* (PtHMAs).

**Figure 2:**
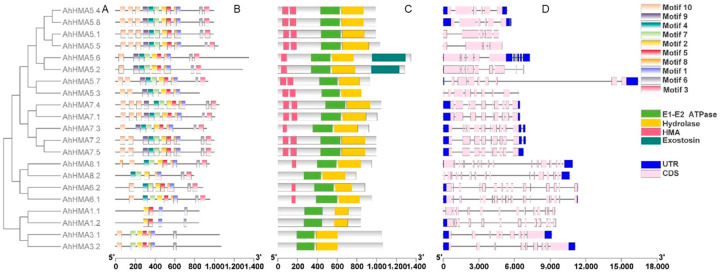
Phylogenetic relationships (**A**), conserved motifs (**B**), domains (**C**), and exon/intron organization (**D**) of AhHMA proteins or genes from peanut. UTR and CDS represent untranslated regions and coding sequences, respectively.

The phylogenetic relationship of 78 HMAs from peanut, *Arabidopsis*, rice, soybean, barrel medic, and black cottonwood was analyzed with the NJ method. These HMA proteins were divided into six groups (I–VI), belonging to two clades representing divalent Zn/Co/Cd/Pb-ATPases (groups I and II) and monovalent Cu/Ag-ATPases (groups III–VI), respectively (Figure 1 and Figure 2A). Of the six primary groups, group VI is the largest one (AhHMAs 5.1–5.8), followed by group V (AhHMAs 7.1–7.5), while the remaining four groups contained two AhHMA members each (Figure 1). Peanut is more closely related to the two legumes (soybean and barrel medic) in terms of *HMA* genes, compared to other plant species.

### 2.3. Conserved Motifs, Domain Architectures, and Models of AhHMA Proteins

A total of ten conserved motifs were identified in AhHMA proteins, and most of them were annotated to be the P-type cation-transporting ATPase according to the InterProScan tool (Figure 2B and Table S1). The motif composition varied among different phylogenetic groups. Groups V and VI had all the ten motifs except AhHMA5.3, while group I contained only four motifs. However, motif numbers, types, and orders were generally similar among members of the same group. All AhHMAs shared two motifs, motif 1 and 2, which contained DKTGT and TGE, respectively. The two motifs might be the characteristic motifs of AhHMA proteins. Motif 9 was specifically distributed in groups V and VI.

All AhHMA proteins contained the typical domains, namely E1–E2 ATPase and hydrolase domains (Figure 2C). Additionally, all members of Cu/Ag-ATPases except AhHMA8.2 contained one or two HMA domains, and AhHMA5.2/5.6 contained an exostosin domain (Figure 2C). No HMA domain was identified in Zn/Co/Cd/Pb-ATPases (groups I and II).

To better understand the function of AhHMA proteins, their sequences were modeled using the SwissModel. As shown in Table 2, the sequence identity ranged from 30.06% to 44.86%, the value of GMQE ranged from 0.41 to 0.68, and the QMEANDisCo global score ranged from 0.56 to 0.70. These data suggested a high quality of 3D protein structure models for AhHMA proteins, which were presented as Figure S3. The 3D-model prediction revealed that AhHMA proteins differed from each other in the 3D structure (Table 2). The best templates of AhHMA1.1/1.2, AhHMA6.1/6.2, and AhHMA8.1/8.2 are 7qc0.1 (Cd translocating P-type ATPase), 7xuk.1 (Cu-transporting ATPase 2), and 4bbj.1 (Cu efflux ATPase), respectively, while 7si3.1 (P-type Cu^+^ transporter) is the best template for the remaining AhHMA members (Table 2).

### 2.4. Exon/Intron Organization and Duplication of AhHMA Genes

All *AhHMA* genes had multiple exons (5–19), and the exon/intron organization widely varied among phylogenetic groups (Figure 2D). The largest number of introns was found in groups III and IV (15–18), followed by group I (12–13), while group VI had the fewest introns (4–8). All orthologs showed exon/intron divergences except *AhHMA3.1*/*3.2*, which resulted from various mechanisms including exon/intron gain/loss (*AhHMA6.1*/*6.2*, *AhHMA7.1*/*7.4*, *AhHMA8.1*/*8.2*), insertion/deletion (*AhHMA1.1*/*1.2*, *AhHMA5.2*/*5.6*, *AhHMA5.4*/*5.8*, *AhHMA7.2*/*7.5*), and exonization/pseudoexonization (*AhHMA5.1*/*5.5*, *AhHMA5.3*/*5.7*, *AhHMA6.1*/*6.2*, *AhHMA8.1*/*8.2*).

**Figure 3:**
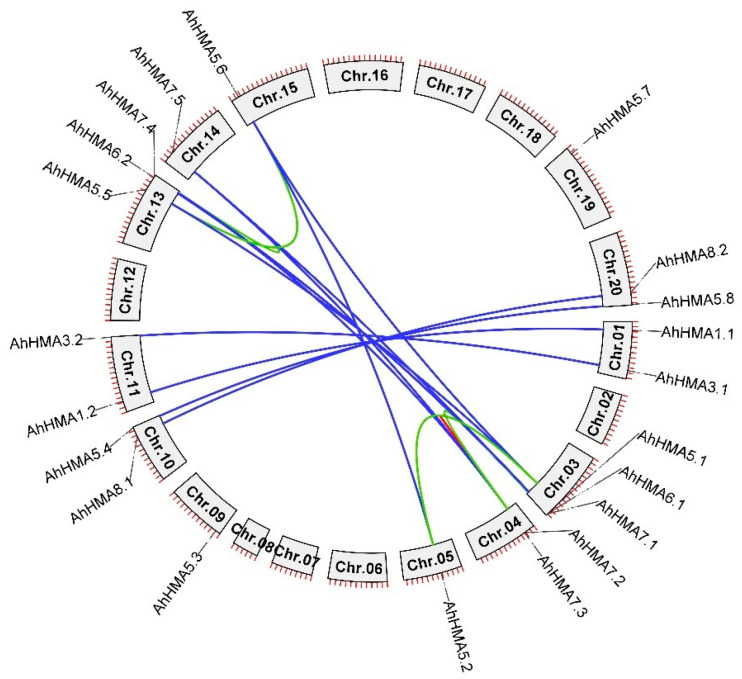
Chromosomal locations and duplications of *AhHMA* genes in peanut. The gene pairs derived from whole-genome duplication, segmental duplication, and tandem duplication are linked by blue, green, and red lines, respectively.

*AhHMA* genes are unevenly distributed among 12 chromosomes in the peanut genome. The subgenomes A (Chr.01-10) and B (Chr.11-20) possess 11 and 10 *AhHMA* genes, respectively (Figure 3). Chromosomes 03 and 13 contain three *AhHMA* genes, chromosomes 01, 04, 10, 11, and 20 contain two *AhHMA* genes, and the remaining five chromosomes (05, 09, 14, 15 and 19) contain only one *AhHMA* gene (Figure 3).

The collinearity analysis revealed that all *AhHMA* genes of peanut underwent gene duplication events (Figure 3). Among the 21 *AhHMA* genes, 20 were detected to undergo whole-genome duplications (WGDs), resulting in 10 gene pairs including *AhHMA1.1/1.2*, *AhHMA3.1/3.2*, *AhHMA5.1/5.5*, *AhHMA5.2/5.6*, *AhHMA5.3/5.7*, *AhHMA5.4/5.8*, *AhHMA6.1/6.2*, *AhHMA7.1/7.4*, *AhHMA7.2/7.5*, and *AhHMA8.1/8.2*. Four gene pairs (*AhHMA5.1/5.2*, *AhHMA5.5/5.6*, *AhHMA7.1/7.2*, and *AhHMA7.4/7.5*) experienced segmental duplication. In addition, one gene pair (*AhHMA7.2/7.3*) was detected to undergo tandem duplication. The *Ka*/*Ks* ratios of duplicated gene pairs ranged from 0.089 to 0.755 (Table 3), indicating that *AhHMA* genes evolved under purifying selection [37]. The divergence time for the 10 whole-genome duplicated gene pairs ranged from 0.77 Mya to 6.03 Mya, which was higher than for the tandem duplicated gene pair (0.58 Mya), but dramatically less than for the four segmental duplicated gene pairs (38.87–82.41 Mya) (Table 3).

### 2.5. Expression Profiles of AhHMA Genes in Different Tissues of Peanut

**Figure 4:**
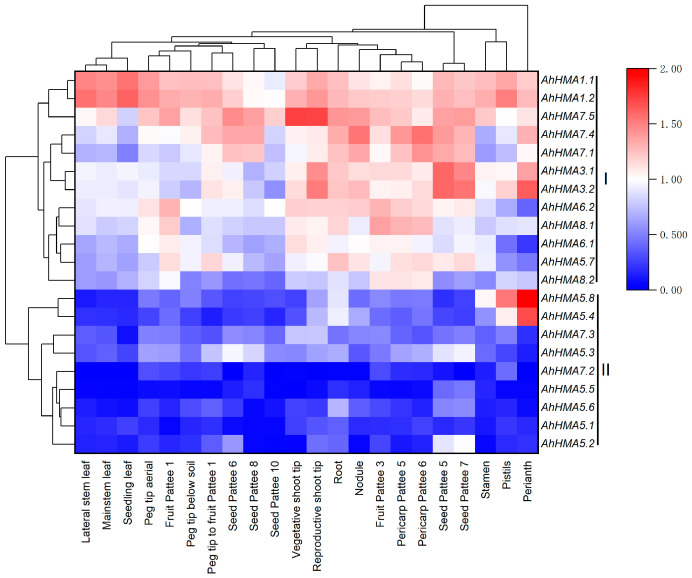
The expression profiles of *AhHMA* genes in different tissues of peanut. Gene expression is expressed in lg(TPM + 1) and Z score normalized. Pattee 1, 3, 5, 6, 7, 8, and 10 represent different developmental stages of peanut pods according to Pattee’ s classification [38].

RNA-seq data revealed that all *AhHMA* genes are expressed in multiple tissues of peanut, and most of them showed a relatively higher expression in roots (Table S2). As presented in Figure 4, 21 *AhHMA* genes were divided into two clusters. Cluster I consists of 12 *AhHMA* genes with high expression levels. Among them, *AhHMA1.1/1.2* and *AhHMA7.5* were highly expressed in all tissues at different development stages, and *AhHMA1.1/1.2* was preferentially expressed in leaves, while *AhHMA7.5* was preferentially expressed in shoot tips. *AhHMA7.1/7.4* was preferentially expressed in nodules, pericarps, and seeds. *AhHMA3.1/3.2* was highly expressed in reproductive shoot tips, perianth, and seeds. Cluster II contains nine genes with low expression levels, including two *AhHMA7* and seven *AhHMA5* genes. Among them, *AhHMA5.4* and *AhHMA5.8* were specifically and highly expressed in perianth and pistil.

### 2.6. Differential Expression of AhHMA Genes in the Root of Two Peanut Cultivars

**Figure 5:**
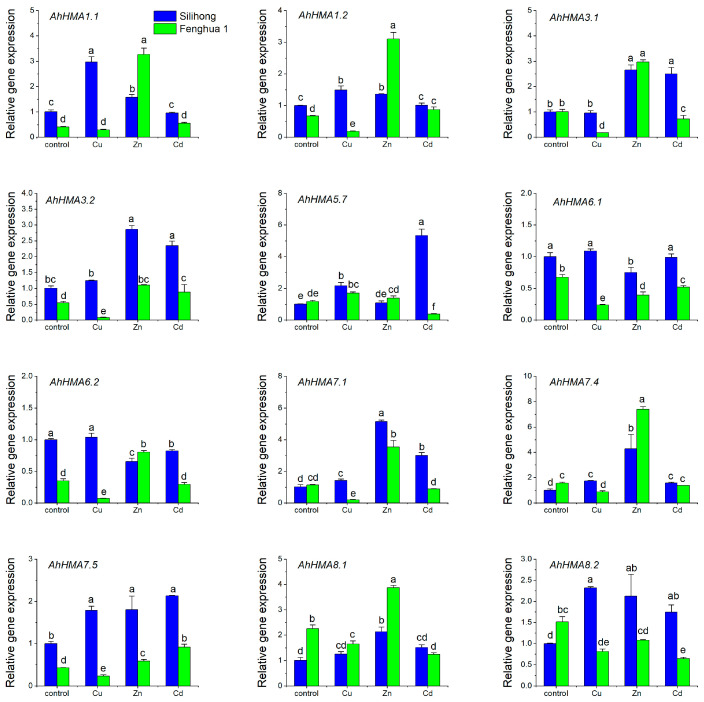
Expression levels of 12 *AhHMA* genes in the root of two peanut cultivars exposed to excess metals for three days. Data (means ± SE, n = 3) sharing the same letter(s) above the error bars are not significantly different at the 0.05 level by the Duncan multiple range test.

To elucidate the responses of *AhHMA* genes to excessive metal stresses, two peanut cultivars differing in Cd accumulation (Fenghua 1 and Silihong) were used for qRT-PCR analysis. As presented in Table S3, exposure of peanut seedlings to Cu, Zn, and Cd for three days did not change the dry weight of roots and shoots. However, the expression of *AhHMA* genes was significantly affected by HM exposure (Figure 5). Excessive Cu increased the expression of *AhHMA5.7* for both cultivars. The expression of the remaining 11 *AhHMA* genes was repressed by excess Cu in Fenghua 1, while it was induced or unaffected in Silihong (Figure 5). Zn induced the expression of *AhHMA1.1*/*1.2*, *AhHMA3.1*/*3.2*, *AhHMA7.1*/*7.4*/*7.5*, and *AhHMA8.1* for both cultivars, while the expression of *AhHMA6.1* was repressed (Figure 5). The expression of *AhHMA6.2* was down-regulated by Zn in Silihong, and up-regulated by Zn in Fenghua 1. The expression of *AhHMA8.2* was induced by Zn in Silihong, but was unaffected in Fenghua 1 (Figure 5). Cd enhanced the expression of *AhHMA3.2* and *AhHMA7.5* for both cultivars, while other genes showed cultivar differences in transcriptional response to Cd stress. The expression of most *AhHMA* genes was induced by Cd in Silihong, while it was repressed or unaffected in Fenghua 1 (Figure 5).

The two cultivars differ from each other in the response of *AhHMA* genes to excessive metal stress (Figure 5). Under the control condition, Silihong showed higher expressions of *AhHMA1.1*, *AhHMA1.2*, *AhHMA3.2*, *AhHMA6.1*, *AhHMA6.2*, and *AhHMA7.5* than Fenghua 1, while the expression of *AhHMA7.4*, *AhHMA8.1*, and *AhHMA8.2* was higher in Fenghua 1 than that in Silihong (Figure 5).

### 2.7. Metal Accumulation and Translocation in Two Peanut Cultivars

**Figure 6:**
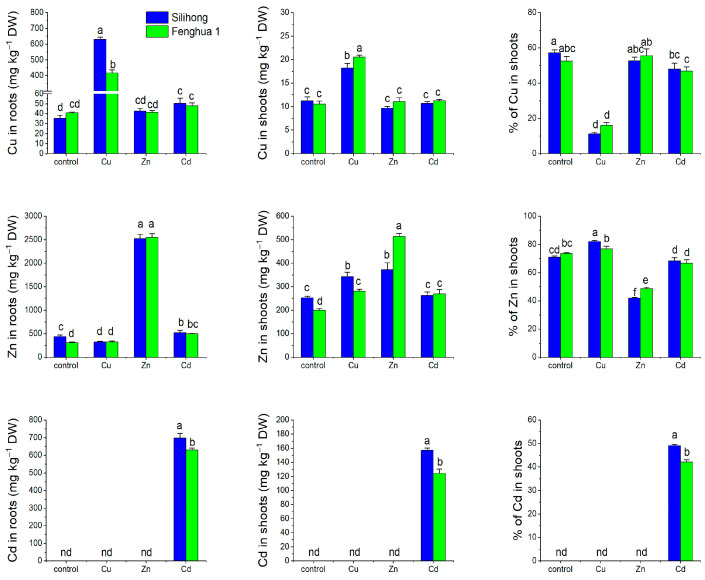
Accumulation and translocation of Cu, Zn, and Cd in two peanut cultivars exposed to excess metals for three days. Data (means ± SE, n = 3) sharing the same letter(s) above the error bars are not significantly different at the 0.05 level by the Duncan multiple range test. nd—not determinable.

Excess Cu and Zn significantly increased their corresponding concentrations in roots and shoots, but reduced the percentage of Cu and Zn in shoots (Figure 6). Cu accumulation and translocation in both peanut cultivars was not affected by Zn. However, Cd exposure increased Cu concentration in the root of Silihong, but reduced the percentage of Cu in shoots. Excess Cu increased Zn concentration in shoots and percentage of Zn in shoots for both cultivars. Cd exposure enhanced root Zn concentration in both cultivars, while an increase of shoot Zn concentration and a reduction of percentage of Zn in shoots was only observed in Fenghua 1 (Figure 6).

The two cultivars differed from each other in metal accumulation and translocation, which is dependent on metal concentrations. Under high concentrations, Silihong exhibited higher Cu concentration in the root, but lower Cu concentration in the shoot compared with Fenghua 1 (Figure 6). Cd accumulation and root-to-shoot translocation were also significantly higher in Silihong than in Fenghua 1. However, Fenghua 1 showed a higher Zn concentration in shoots and higher percentage of Zn in shoots than Silihong under excess Zn condition (Figure 6).

To determine whether *AhHMA* genes were involved in the accumulation of Cu and Zn in peanut plants, a correlation analysis was performed on the expression of *AhHMA* genes in the roots and the accumulation of Cu and Zn in plants. As shown in Table 4, the expression of *AhHMA3.1* and *AhHMA7.1* was negatively correlated with Cu concentration in shoots, but positively correlated with the percentage of Cu in shoots. The expression of *AhHMA1.2*, *AhHMA3.1*/*3.2*, *AhHMA7.1*/*7.4*, and *AhHMA8.1* was positively correlated with Zn concentrations in roots and/or shoots, but negatively correlated with the percentage of Zn in shoots (Table 4).

## 3. Discussion

### 3.1. WGD Expanded the Number of AhHMA Genes in Peanut

Genome-wide identification of HMA proteins has been extensively performed in diverse plant species, including *Arabidopsis* [6], rice [7], soybean [39], barrel medic [40], canola (*Brassica napus*) [41], flax (*Linum usitatissimum*) [42], and black cottonwood [43]. However, little is known about the HMA family in peanut, limiting understanding of the molecular mechanisms of metal homeostasis. Herein, we identified 21 *AhHMA* genes in peanut genome, and all of them are multicopy genes (Figure 1). The number of *HMA* genes in peanut is higher than that in almost all the reported plant species, such as *Arabidopsis* (8) [6], rice (9) [7], black cottonwood (12) [43], soybean (20) [39], barrel medic (9) [40], and flax (12) [42], but less than that in canola, which contains 31 *HMA* genes [40]. A large number of genes in the peanut genome have been reported in other gene families, such as *AhMTP*s [30], *AhZIP*s [31], and *AhOPT*s [33].

Peanut, as an allotetraploid species, has A and B subgenomes derived from its diploid progenitors, *A. duranensis* (A genome) and *A. ipaensis* (B genome), respectively [35]. WGD events have occurred at least three times in the evolutionary history of peanut, together with allopolyploidization [44]. In the current study, collinearity analysis revealed that all *AhHMA* genes of peanut underwent gene duplication events, resulting in 10 pairs of WGD orthologs (Figure 3). It is likely that the WGD during allopolyploidization expanded the number of *AhHMA* genes [33], resulting in multicopy genes. Compared with the B subgenome, the A subgenome has an additional *AhHMA* gene (*AhHMA7.3*) resulting from tandem duplication. These differences confirmed that the peanut subgenomes evolved asymmetrically [44], with the A subgenome undergoing more gene duplication than the B subgenome.

### 3.2. Conservation and Divergence of AhHMA Proteins in Peanut

In agreement with previous studies [5,39,40], AhHMA proteins were classified into six groups (I-VI), belonging to two substrate-specific clades (Zn/Co/Cd/Pb-ATPases and Cu/Ag-ATPases) (Figure 1). The four members of the Zn/Co/Cd/Pb-ATPases contain eight TMDs, fewer motifs (four–seven motifs), and no HMA domains. In contrast, Cu/Ag-ATPases contain six to nine TMDs, more motifs (eight–ten), and one or two HMA domains (Figure 2).

Multiple sequence alignment revealed that all AhHMA proteins contain conserved motifs of P_1B_-ATPases including TGE, DKTGT, CPC/SPC, GDGxNDxP, HP, and PxxK, except AhHMA5.3/5.7 (without GDGxNDxP or PxxK) and AhHMA6.1/6.2 (without HP) (Figure S2). These motifs might play essential roles in the metal transport of AhHMA proteins in peanut. Mutation of the TGE motif severely compromises the transport ability of *Arabidopsis ran1-1* mutant [15]. The DKTGT motif contains a phosphorylatable aspartate (D), and mutations of the D residue severely inhibit the transport capabilities of AtHMA3 and AtHMA4 [5,45,46]. The CPC/SPC motif might play a role in metal transport, and mutations of this motif eliminate transport function in AtHMA4 [47]. D residues in the GDGxNDxP motif might bind magnesium [5,48]. The histidine in the HP motif is important for nucleotide coordination in the N-domain of ATP7B, together with a nearby conserved glutamate [49]. The lysine (K) residue in the PxxK motif might interact with the oxygen of the phosphate that is transferred from ATP [50]. The lack of GDGxNDxP in AhHMA5.3, PxxK in AhHMA5.7, and HP in AhHMA6.1/6.2 suggests that these proteins might have evolved unique metal transport mechanisms.

AhHMA proteins were well modeled with four kinds of 3D model templates such as 7qc0.1 (Cd translocating P-type ATPase), 7xuk.1 (Cu-transporting ATPase 2), 7si3.1 (P-type Cu+ transporter), and 4bbj.1 (Cu efflux ATPase) (Table 2). The 7qc0.1 is the crystal structure of the P_1B-4_-ATPase (sCoaT) from *Sulfitobacter* sp. NAS14-1, which was shown to transport metal ions such as Zn^2+^, Cd^2+^, and Co^2+^ [51]. The 7si3.1 and 7xuk.1 represent the cryo-electron microscopy structure of ATP7B, a P-type ATPase that exports cytosolic copper from frogs and humans, respectively [52,53]. The 4bbj.1 shows a crystal structure of Cu^+^-ATPase from *Legionella pneumophila* (LpCopA), which has a unique copper transport pathway across the membrane [54]. Structural analysis indicates that AhHMAs might transport multiple metal ions in different pathways.

### 3.3. Divergence of Duplicated AhHMA Genes in Peanut

Gene duplication is the major source of novel genes that foster the acquirement of novel functions [55]. However, duplicated genes are usually functionally redundant and most of them would quickly pseudogenize and get lost [56,57]. To avoid gene loss, duplicated genes should firstly reduce their expression for hypofunctionalization or dosage sharing [55,56,58]. In the present study, nine *AhHMA* genes exhibited low expression in all peanut tissues under normal condition, and all of them are multicopy genes with a copy number larger than two. It seems that the higher the copy number, the higher the percentage of lowly expressed genes. Similar results have been reported in the *MTP*, *ZIP*, and *OPT* families of peanut [30,31,33]. These results, in agreement with Qian [56], indicate that the reduced expression might be an important mechanism for maintaining duplicates and their functional redundancy.

If duplicated genes survive, they can split functions (subfunctionalization) or generate a new function (neofunctionalization) by diverging in both the regulatory and coding regions [55,58,59]. Thus, exon/intron organization can provide additional evidence to survey the functional divergence of duplicated genes. In the present study, all duplicated genes showed exon/intron divergences except *AhHMA3.1*/*3.2*. These divergences, according to Xu et al. [59], resulted from various mechanisms including exon/intron gain/loss (*AhHMA6.1*/*6.2*, *AhHMA7.1*/*7.4*, and *AhHMA8.1*/*8.2*), insertion/deletion (*AhHMA1.1*/*1.2*, *AhHMA5.2*/*5.6*, *AhHMA5.4*/*5.8*, and *AhHMA7.2*/*7.5*), and exonization/pseudoexonization (*AhHMA5.1*/*5.5*, *AhHMA5.3*/*5.7*, *AhHMA6.1*/*6.2*, and *AhHMA8.1*/*8.2*). The data of divergence time indicate that segmental duplications (38.87–82.41 Mya) might have occurred before the allopolyploidization, while the tandem duplication (0.58 Mya) occurred after the WGD (0.77–6.03 Mya) (Table 3).

### 3.4. Potential Role of AhHMA Genes in Metal Transport in Peanut

Our results showed that the expression of *AhHMA1.1*/*1.2*, *AhHMA3.1*/*3.2*, *AhHMA7.1*/*7.4*, and *AhHMA8.1* induced by Zn stress for both cultivars, which is significantly correlated with Zn accumulation and translocation (Figure 5 and Table 4). These findings indicate that these genes might be involved in Zn accumulation and translocation in peanut plants. *AtHMA1*, a homologous gene of *AhHMA1.1*/*1.2* in *Arabidopsis*, has been shown to confer Zn detoxification by reducing the Zn content in the chloroplast [12]. Several homologous genes of *AhHMA3.1*/*3.2*, such as *AtHMA2/4* and *AtHMA3* in *Arabidopsis* and *OsHMA2* and *OsHMA3* in rice, were confirmed to be involved in Zn distribution and translocation. AtHMA2/4 and OsHMA2 are responsible for xylem loading of Zn for long-distance translocation [17,18,19,21,22]. AtHMA3 and OsHMA3 are tonoplast-localized transporters participating in Zn detoxification by vacuolar sequestration [20,23,24]. *AhHMA7* genes were closely clustered with *OsHMA9*, which is localized to the PM and is responsible for Zn efflux from the cytoplasm [27].

Most *AhHMA* genes tested in this study showed cultivar-specific transcriptional responses to Cu exposure in peanut roots. For example, the expression of *AhHMA3.1* and *AhHMA7.1* was reduced by Cu stress in Fenghua 1, but not in Silihong. Moreover, the expression of *AhHMA3.1* and *AhHMA7.1* was significantly correlated with shoot Cu concentrations and the percentage of Cu in shoots. AtHMA7 has been confirmed to play a crucial role in delivering Cu^+^ across post-Golgi membranes to increase ethylene-binding activity [15,16]. Additionally, we found that the expression of *AhHMA5.7* was increased by Cu stress for both cultivars. AtHMA5 was shown to confer Cu detoxification in roots and/or xylem loading for root-to-shoot Cu translocation [13,14]. OsHMA5 is involved in loading Cu to the xylem for root-to-shoot translocation [26]. OsHMA4, a homologous gene of *AhHMA5.7*, functions to sequester Cu into root vacuoles, preventing Cu accumulation in rice grain [25]. Unfortunately, correlations between the expression of *AhHMA5.7* and Cu accumulation and translocation were not significant. Whether *AhHMA3.1*, *AhHMA7.1*, and *AhHMA5.7* are involved in Cu translocation in peanut plants merits further study.

The response of *AhHMA* gene expression in peanut roots to Cd exposure is also dependent on cultivars. The expression of *AhHMA3.2* and *AhHMA7.5* was induced by Cd for both cultivars. Although there is a lack of correlation data, the two genes showed higher expression levels in Silihong (high Cd accumulation cultivar) compared with Fenghua 1 (low Cd accumulation cultivar). These data indicate that *AhHMA3.2* and *AhHMA7.5* might be responsible for Cd transport in peanut plants. *AtHMA3* and *OsHMA3* have been demonstrated to participate in Cd detoxification by vacuolar sequestration [20,23,24]. Similarly, TcHMA3 from *Thlaspi caerulescens* has also been confirmed to be a tonoplast-localized transporter, which is responsible for sequestration of Cd into the leaf vacuoles [60]. GmHMA3 sequesters Cd to the root endoplasmic reticulum to limit translocation to the stems in soybean [61]. In addition, *AhHMA3.1*, *AhHMA5.7*, *AhHMA7.1*, and *AhHMA8.2* exhibit higher expression in Silihong than that in Fenghua 1. Further studies are required to determine whether these genes are related to higher cadmium accumulation in Silihong.

## 4. Materials and Methods

### 4.1. Plant Growth and Treatment

Two peanut cultivars differing in Cd accumulation, Fenghua 1 (lower Cd cultivar) and Silihong (higher Cd cultivar), were used in this study [36]. Seeds were surface sterilized with 5% sodium hypochlorite and then sown in vermiculite for germination. Three days after germination, seedlings with uniform size were transferred to polyethylene pots for hydroponic culture (three seedlings per cultivar in each pot). The formula of the nutrient solution (pH 5.8) is as follows: 5 mM KNO_3_, 5 mM Ca(NO_3_)_2_, 1 mM MgSO_4_, 1 mM KH_2_PO_4_, 4.5 mM MnCl_2_, 50 mM H_3_BO_3_, 0.3 mM CuSO_4_, 3.8 mM ZnSO_4_, 50 mM FeEDTA and 0.1 mM (NH_4_)_6_Mo_7_O_24_. The 14-d-old seedlings were treated with HMs for three days by adding an additional 30 µM CdCl_2_, 50 µM CuSO_4_, or 500 µM ZnSO_4_ to the nutrient solution, respectively. The non-treated plants serve as the control. Three biological replicates were prepared for each HM treatment. Plants were cultured in a growth chamber under conditions of a 16 h light/8 h dark photoperiod, irradiance of 805 ± 73 mmol m^−2^ s^−1^, day/night temperatures of 26.5 ± 1.4/22.1 ± 1.6 °C and relative humidity of 56 ± 8%. During the cultivation period, the nutrient solution is replaced twice a week, and the pot is randomly moved to avoid positional effects. At the end of HM treatment, fresh root tissues were sampled, immediately frozen in liquid nitrogen, and stored at −80 °C for qRT-PCR analysis.

### 4.2. Metal Determination

The harvested roots and shoots were oven-dried, weighed, and ground into powder. Root and shoot powders were digested with the mixed solution of HNO_3_-HClO_4_ (3:1, *v*/*v*). Metal concentrations were determined by flame atomic absorbance spectrometry (WFX-210, Beijing Rayleigh Analytical Instrument Company, China). A certified standard reference material (peach leaves, GBW-08501) was used for the quality control of metal determination. The recovery ratio ranged from 93% to 98%, from 95% to 99%, and from 92% to 97% for Cd, Cu, and Zn, respectively.

### 4.3. Identification of HMA Genes in Peanut and Phylogenetic Analysis

Protein sequences of *Arabidopsis* (AtHMA1-8) and rice (OsHMA1-9) were obtained from the phytozome database (https://phytozome-next.jgi.doe.gov, accessed on 8 November 2022). Using these sequences as queries, BLASTP was carried out against the peanut genome on PeanutBase (https://peanutbase.org, accessed on 8 November 2022) with an E value < 10^−10^. Non-redundant sequences were examined with the hmmscan tool (https://www.ebi.ac.uk/Tools/hmmer/search/hmmscan, accessed on 8 November 2022). After removing sequences without E1–E2 ATPase (PF00122) and hydrolase (PF00702) domains, the remaining protein sequences were aligned with those of AtHMAs and OsHMAs using the ClustalW in the MEGA-X program (v10.2.6). The alignment file was used to construct a phylogenetic neighbor-joining (NJ) tree using the Poisson model with 1000 bootstrap replicates. The proteins clustered with AtHMAs and OsHMAs were defined as putative AhHMAs in peanut. To investigate the phylogenetic relationships of AhHMAs, another phylogenetic tree was constructed with the HMA protein sequences of peanut, *Arabidopsis*, rice, soybean (*Glycine max*), barrel medic (*Medicago truncatula*), and black cottonwood (*Populus trichocarpa*). The phylogenetic tree was displayed and modified using an online software iTOL (https://itol.embl.de/itol.cgi, accessed on 10 November 2022). Unless otherwise specified, all bioinformatics analyses are conducted under default settings.

### 4.4. Physicochemical and Structural Characteristics of AhHMA Proteins

Molecular weight (MW), grand average of hydropathicity (GRAVY), isoelectric points (pI), instability index, and aliphatic index of AhHMA proteins were analyzed using the ProtParam tool (https://web.expasy.org/protparam/, accessed on 15 October 2023) [62]. TMDs were predicted using TOPCONS (http://topcons.net/, accessed on 15 October 2023) [63]. Subcellular localization of AhHMA proteins was predicted by ProtComp v. 9.0 (http://www.softberry.com/berry.phtml?topic=protcomppl&group=programs&subgroup=proloc, accessed on 20 October 2023). The conserved motifs and domains were examined using the MEME v. 5.3.3 (https://meme-suite.org/meme/tools/meme, accessed on 20 November 2022) [64] and Pfam tool (http://pfam.xfam.org/search#tabview=tab1, accessed on 20 November 2022) [65], respectively. Homology-modeled 3D structures of AhHMA proteins were predicted using the SwissModel (https://swissmodel.expasy.org/, accessed on 20 October 2023) [66]. All bioinformatics analyses are conducted under default settings.

### 4.5. Exon/intron Structure, Duplication and Ka/Ks of AhHMA Genes

Gene Structure Display Server (GSDS v. 2.0) (http://gsds.gao-lab.org/, accessed on 20 November 2022) was used for determining the exon/intron structure of *AhHMA* genes [67]. Gene collinearity and *Ka*/*Ks* (substitution ratios of nonsynonymous (*Ka*) to synonymous (*Ks*)) were analyzed by One Step MCScanX and simple *Ka*/*Ks* calculator (NJ) integrated in TBtools software, respectively [68]. Gene structure and duplication events were visualized using TBtools software [68]. The *Ks* value was used to calculate the divergence time of duplicated *AhHMA* gene pairs with the equation T = *Ks*/2λ, where λ = 8.12 × 10^−9^ for peanut [34].

### 4.6. Expression Profiles of AhHMA Genes in Different Tissues

Expression profiles of *AhHMA* genes in different tissues at different developmental stages were identified using RNA-seq data of cv. Tifrunner was obtained from PeanutBase (https://www.peanutbase.org/gene_expression/atlas, accessed on 1 November 2022) [38]. The expression of *AhHMA* genes was normalized and represented in TPM (Transcripts Per kilobase of exon model per Million mapped reads), and lg(TPM + 1) was used to construct the heatmap diagram by TBtools [68].

### 4.7. RNA Extraction and qRT-PCR

Total RNA extraction, cDNA strand synthesis, and qRT-PCR analysis are followed according to the method described previously [32]. Peanut *Ah60S* was used as an internal control to calculate gene expression levels. Sequences of the specific primer for 12 *AhHMA*s and *Ah60S* are listed in Table S4. The relative gene expression was calculated using the 2^−ΔΔCT^ method with three biological replicates. Three technical replicates were performed for each biological replicate to ensure the accuracy of the results.

### 4.8. Statistical Analysis

Data of metal accumulation and qRT-PCR were expressed as mean ± standard error (SE) of three replicates per treatment/cultivar. One-way analysis of variance (ANOVA) was used to determine significant difference among various treatments/cultivars followed by Duncan’s Multiple Range Test at a probability level of 5%. Pearson’s correlation analysis was performed to detect the relationship between metal accumulation and expression of *AhHMA* genes. All statistical analysis was conducted using IBM SPSS Statistics v. 22 (IBM, New York, NY, USA).

## 5. Conclusions

Here, a total of 21 *AhHMA* genes were identified in the peanut genome, which were divided into six groups (I–VI), belonging to two clades (Zn/Co/Cd/Pb-ATPases and Cu/Ag-ATPases). Among them, 20 genes underwent WGD that resulted in 10 paralogous gene pairs. Most AhHMA members within the same clade or group generally have a similar protein structure. However, exon/intron organization showed significant divergence even between duplicated gene pairs. Most *AhHMA* genes are preferentially expressed in roots, shoots, and reproductive tissues, indicating possible roles in metal uptake and their transport to shoots and reproductive tissues. The induction of *AhHMA1.1*/*1.2*, *AhHMA3.1*/*3.2*, *AhHMA7.1*/*7.4*, and *AhHMA8.1* under Zn stress and their significant correlation with Zn accumulation indicate that these genes might be involved in Zn transport in peanut plants. Additionally, *AhHMA3.2*, and *AhHMA7.5* might be involved in Cd transport. Our findings provide clues to further characterize the functions of *AhHMA* genes in metal uptake and translocation in peanut plants, which is great of importance for screening or breeding cultivars for safe peanut production in heavy metal-contaminated soil.

## Figures and Tables

**Table 1:** Physicochemical properties and subcellular localization of the 21 *AhHMA* genes and corresponding proteins identified in peanut.

Gene Name	Gene ID	Gene Length (bp)	CDS (bp)	Aa ^a^	MW ^b^ (kDa)	Instability	Aliphatic Index	GRAVY ^c^	pI ^d^	No. of TMD ^e^	Location
*AhHMA1.1*	Arahy.VMCJ1G	9517	2541	91.92	846	37.86	102.86	0.121	7.86	8	Chl. ^f^
*AhHMA1.2*	Arahy.FTH7SR	9482	2523	91.23	840	37.43	102.32	0.093	7.5	8	Chl.
*AhHMA3.1*	Arahy.NH81W7	9130	3159	114.12	1052	37.96	88.34	−0.211	6.25	8	PM ^g^
*AhHMA3.2*	Arahy.6QFK0R	11,108	3198	115.79	1065	35.17	87.62	−0.215	6.26	8	PM
*AhHMA5.1*	Arahy.C06U18	4665	2973	107.41	990	36.52	100.37	0.138	6.16	9	PM
*AhHMA5.2*	Arahy.1HUL85	6784	3855	142.51	1284	38.21	96.36	−0.09	6.19	7	PM
*AhHMA5.3*	Arahy.694CK1	11,328	2541	91.90	846	36.14	102.87	0.155	5.73	6	PM
*AhHMA5.4*	Arahy.9DR5PQ	5362	2979	107.31	992	34.58	100.77	0.146	5.95	9	PM
*AhHMA5.5*	Arahy.NXU65W	4972	3111	113.03	1036	35.08	100.89	0.137	7.25	9	PM
*AhHMA5.6*	Arahy.NB9ZER	7292	4041	149.65	1346	37.40	97.86	−0.073	6.94	8	PM
*AhHMA5.7*	Arahy.3M9T3M	16,396	2805	100.97	934	37.39	105.61	0.245	5.53	8	PM
*AhHMA5.8*	Arahy.LQF816	5780	2979	107.45	992	35.35	100.18	0.128	5.96	9	PM
*AhHMA6.1*	Arahy.8T0CRQ	11,318	2862	101.18	953	36.11	101.19	0.192	8.48	8	Chl.
*AhHMA6.2*	Arahy.KT5IG2	11,336	2649	93.68	882	36.93	98.84	0.133	8.88	8	Chl.
*AhHMA7.1*	Arahy.H8X8VQ	6457	3018	107.24	1005	30.82	104.75	0.314	5.14	8	PM
*AhHMA7.2*	Arahy.XIB6CP	6910	2766	98.77	921	34.05	105.1	0.28	5.53	8	PM
*AhHMA7.3*	Arahy.GR9FC3	6947	2994	106.71	997	34.58	105.79	0.299	5.12	8	PM
*AhHMA7.4*	Arahy.PPR7D4	6457	3147	112.04	1048	30.85	106.68	0.313	5.43	8	PM
*AhHMA7.5*	Arahy.8Q2P9V	6759	2994	106.65	997	34.49	105.4	0.296	5.09	8	PM
*AhHMA8.1*	Arahy.ALH963	10,892	2853	101.55	950	39.05	106.11	0.162	5.95	8	Chl.
*AhHMA8.2*	Arahy.39IB73	10,646	2379	84.17	792	32.40	106.87	0.224	5.15	8	Chl.

^a^ amino acid number, ^b^ molecular weight, ^c^ grand average of hydropathicity, ^d^ isoelectric points, ^e^ transmembrane domain, ^f^ Chloroplast, ^g^ Plasma membrane.

**Table 2:** The best templates of peanut AhHMA proteins selected from the SwissModel template library for building 3D structure models.

Protein Name	Template	Sequence Identity (%)	Coverage	GMQE	QMEANDisCo Global	Description
AhHMA1.1	7qc0.1	0.3214	A169-812	0.48	0.62 ± 0.05	Cadmium translocating P-type ATPase
AhHMA1.2	7qc0.1	0.3214	A163-806	0.48	0.62 ± 0.05	Cadmium translocating P-type ATPase
AhHMA3.1	7si3.1	0.3006	A11-702	0.42	0.62 ± 0.05	P-type Cu(+) transporter
AhHMA3.2	7si3.1	0.3006	A11-702	0.41	0.62 ± 0.05	P-type Cu(+) transporter
AhHMA5.1	7si3.1	0.4293	A128-976	0.62	0.65 ± 0.05	P-type Cu(+) transporter
AhHMA5.2	7si3.1	0.4272	A31-874	0.45	0.65 ± 0.05	P-type Cu(+) transporter
AhHMA5.3	7si3.1	0.3942	A117-845	0.64	0.67 ± 0.05	P-type Cu(+) transporter
AhHMA5.4	7si3.1	0.4264	A121-970	0.65	0.70 ± 0.05	P-type Cu(+) transporter
AhHMA5.5	7si3.1	0.4292	A128-1021	0.6	0.65 ± 0.05	P-type Cu(+) transporter
AhHMA5.6	7si3.1	0.4486	A28-869	0.42	0.63 ± 0.05	P-type Cu(+) transporter
AhHMA5.7	7si3.1	0.4192	A94-927	0.68	0.69 ± 0.05	P-type Cu(+) transporter
AhHMA5.8	7si3.1	0.4247	A121-970	0.64	0.69 ± 0.05	P-type Cu(+) transporter
AhHMA6.1	7xuk.1	0.3249	A136-934	0.46	0.56 ± 0.05	Copper-transporting ATPase 2
AhHMA6.2	7xuk.1	0.3276	A136-855	0.51	0.61 ± 0.05	Copper-transporting ATPase 2
AhHMA7.1	7si3.1	0.3885	A134-993	0.62	0.66 ± 0.05	P-type Cu(+) transporter
AhHMA7.2	7si3.1	0.4012	A44-910	0.6	0.62 ± 0.05	P-type Cu(+) transporter
AhHMA7.3	7si3.1	0.3811	A126-986	0.62	0.66 ± 0.05	P-type Cu(+) transporter
AhHMA7.4	7si3.1	0.3862	A134-1037	0.58	0.63 ± 0.05	P-type Cu(+) transporter
AhHMA7.5	7si3.1	0.384	A126-985	0.61	0.65 ± 0.05	P-type Cu(+) transporter
AhHMA8.1	4bbj.1	0.3687	A285-942	0.46	0.66 ± 0.05	Copper efflux ATPase
AhHMA8.2	4bbj.1	0.3672	A149-784	0.52	0.65 ± 0.05	Copper efflux ATPase

**Table 3:** *Ka*/*Ks* analysis of all gene duplication pairs for *AhHMA* genes.

Gene Pairs	Duplicate Type	*Ka* ^a^	*Ks* ^b^	*Ka*/*Ks* ^c^	Positive Selection	Divergence Time (Mya)
*AhHMA5.1*/*5.2*	Segmental	0.150	1.338	0.112	No	82.41
*AhHMA5.5*/*5.6*	Segmental	0.154	1.293	0.119	No	79.60
*AhHMA7.1*/*7.2*	Segmental	0.099	0.631	0.157	No	38.87
*AhHMA7.4*/*7.5*	Segmental	0.093	0.716	0.130	No	44.10
*AhHMA7.2*/*7.3*	Tandem	0.007	0.009	0.755	No	0.58
*AhHMA1.1*/*1.2*	Whole-genome	0.002	0.024	0.089	No	1.45
*AhHMA3.1*/*3.2*	Whole-genome	0.010	0.043	0.228	No	2.68
*AhHMA5.1*/*5.5*	Whole-genome	0.006	0.030	0.208	No	1.85
*AhHMA5.2*/*5.6*	Whole-genome	0.014	0.040	0.343	No	2.46
*AhHMA5.3*/*5.7*	Whole-genome	0.024	0.064	0.384	No	3.92
*AhHMA5.4*/*5.8*	Whole-genome	0.011	0.020	0.565	No	1.25
*AhHMA6.1*/*6.2*	Whole-genome	0.002	0.012	0.122	No	0.77
*AhHMA7.1*/*7.4*	Whole-genome	0.005	0.019	0.256	No	1.17
*AhHMA7.2*/*7.5*	Whole-genome	0.016	0.039	0.399	No	2.41
*AhHMA8.1*/*8.2*	Whole-genome	0.048	0.098	0.489	No	6.03

^a^ The number of nonsynonymous substitutions per nonsynonymous site, ^b^ the number of synonymous substitutions per synonymous site, ^c^ *Ka*/*Ks* ratios.

**Table 4:** Correlation coefficient (n = 24) between the expression of AhHMA genes in the roots and the accumulation of Cu and Zn in plants of Fenghua 1 and Silihong.

Gene Expression	[Cu]_root_	[Cu]_shoot_	% of Cu in Shoots	[Zn]_root_	[Zn]_shoot_	% of Zn in Shoots
*AhHMA1.1*	0.283	0.061	−0.087	0.534 **	0.798 **	−0.292
*AhHMA1.2*	−0.155	−0.278	0.313	0.721 **	0.848 **	−0.560 **
*AhHMA3.1*	−0.471 *	−0.589 **	0.546 **	0.785 **	0.593 **	−0.776 **
*AhHMA3.2*	−0.291	−0.504 *	0.331	0.514 *	0.245	−0.579 **
*AhHMA5.7*	0.091	0.037	−0.110	−0.188	−0.120	0.179
*AhHMA6.1*	0.097	−0.206	0.032	−0.248	−0.248	0.222
*AhHMA6.2*	0.050	−0.209	0.156	0.189	0.325	−0.082
*AhHMA7.1*	−0.382	−0.523 **	0.430 *	0.851 **	0.586 **	−0.845 **
*AhHMA7.4*	−0.276	−0.336	0.340	0.895 **	0.890 **	−0.762 **
*AhHMA7.5*	0.066	−0.221	−0.011	0.089	0.012	−0.135
*AhHMA8.1*	−0.295	−0.277	0.325	0.728 **	0.683 **	−0.611 **
*AhHMA8.2*	0.285	−0.022	−0.145	0.137	0.037	−0.061

* *p* < 0.05, ** *p* < 0.01.

## Data Availability

RNA-seq data are obtained from PeanutBase which is submitted by Clevenger et al. [38]. Raw expression data are available from NCBI’s GEO database (https://www.ncbi.nlm.nih.gov/geo/query/acc.cgi?acc=GSE71357, accessed on 1 November 2022), accession number: GSE71357.

## Supplementary Materials

The following supporting information can be downloaded at: https://www.mdpi.com/article/10.3390/ijms25010613/s1.
