# Supplementary material for: Genome-Wide Identification and Expression Profiling of Heavy Metal ATPase (HMA) Genes in Peanut: Potential Roles in Heavy Metal Transport
Source: Int J Mol Sci. 2024 Jan 3;25(1):613. doi: 10.3390/ijms25010613 (PMC10779257; doi:10.3390/ijms25010613)
Supplement: Supplementary file 1 [file ijms-25-00613-s001.zip › supplementary figures.pdf]

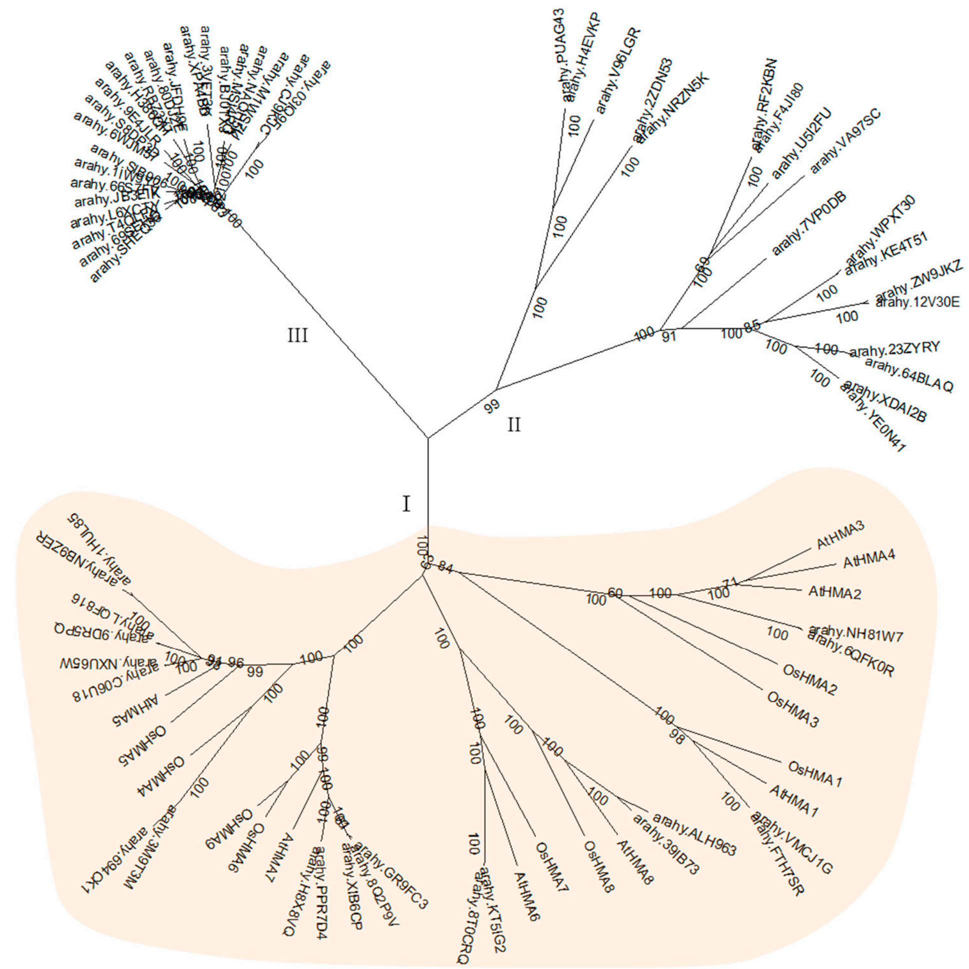

Figure S1. Screening HMAs from candidate sequences identified by a BLASTP search against the peanut genome using phylogenetic analysis with AtHMAs from *Arabidopsis* and OsHMAs from rice. A total of 21 protein sequences were identified to be putative AhHMA members (cluster I).

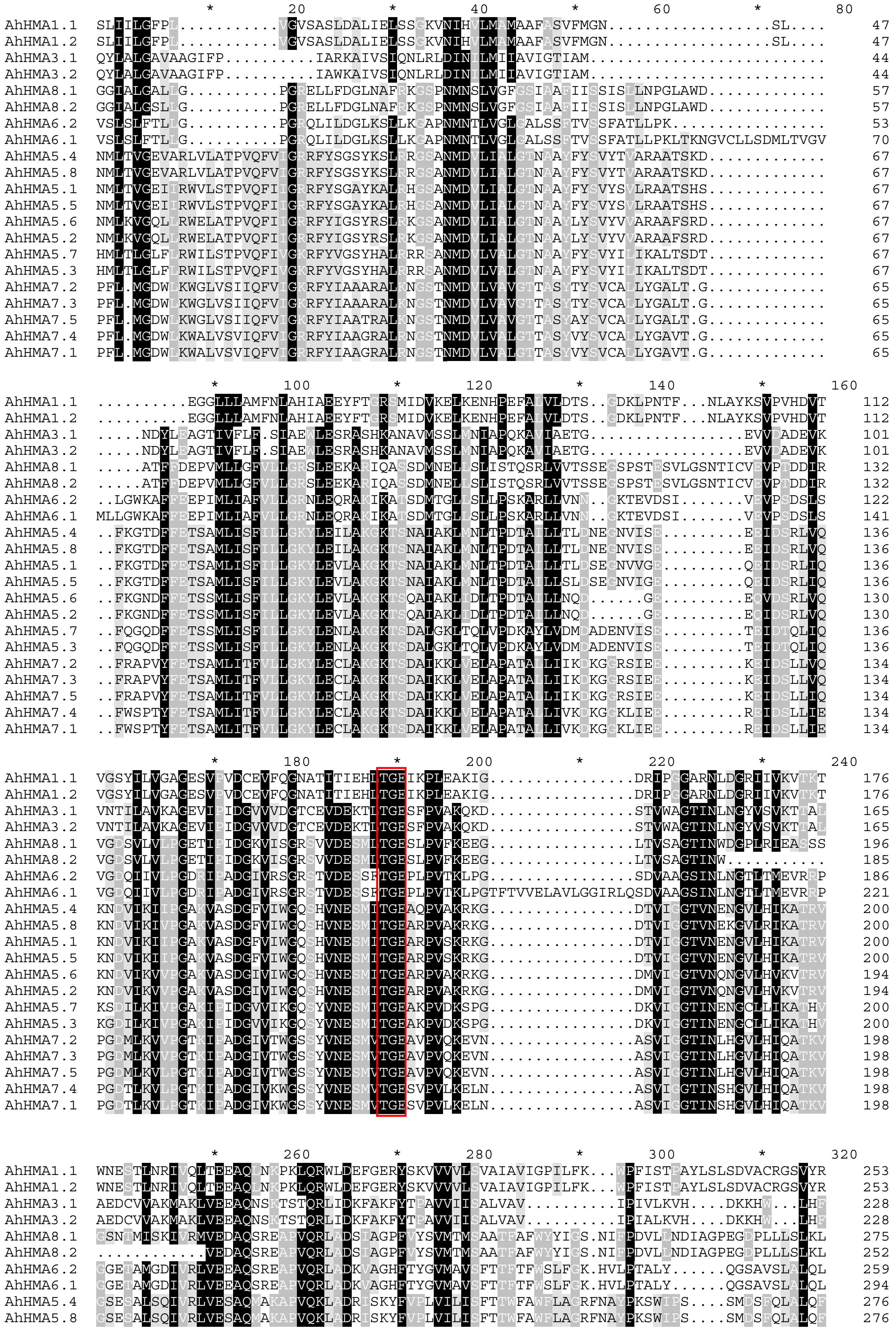

```
AhHMA5.1   GSESALSQIVRLVESAQMAKAPVQRFADRISKYFVPLVILISFSTWLSWFLAGRFHAYPDSWIPS....SMDSFELALQF   276
AhHMA5.5   GSESALSQIVRLVESAQMAKAPVQKFADRISKYFVPLVILISFSTWLSWFLAGRFHAYPESWIPS....SMDSFELALQF   276
AhHMA5.6   GSESALSQIVRLVESAQMAKAPVQKLADHISKYFVPLVILLSFSTWIAWYLAGKLHAYPKSWIPS....SMNSFELALQF   270
AhHMA5.2   GSESALSQIVRLVESAQMAKAPVQKLADHISKYFVPLVILLSFSTWIAWYLAGKLHAYPKSWIPS....SMNSFELALQF   270
AhHMA5.7   GSDTALSQIVQLVETAQLAKAPVQKLADHISRVFVPLVVVAAFLTWMGWFVPGEVGLYPRHWIPK....AMNAFELALQF   276
AhHMA5.3   GSDTALSQIVQLVETAQLAKAPVQKLADHISRVFVPLVVVAAFLTWMGWFVPGEVGLYPRHWIPK....AMNAFELALQF   276
AhHMA7.2   GSDTVLSQIISLVETAQMSKAPIQKFADYVASIFVPTVVSLALLTFFSWYGAGSIGAYPEEWLPE....NGNHFVFALMF   274
AhHMA7.3   GSDTVLSQIISLVETAQMSKAPIQKFADYVASIFVPTVVSLALLTFFSWYGAGSIGAYPEEWLPE....NGNHFVFALMF   274
AhHMA7.5   GSDTVLSQIISLVETAQMSKAPIQKFADYVASIFVPTVVSLALLTFFSWYGAGSVGAYPEEWLPE....NGNHFVFALMF   274
AhHMA7.4   GSDTVLSQIISLVETAQMSKAPIQKFADYVASIFVPTVVALSLLTFL..YTAGALEAYPKEWLPE....NGNHFVFALMF   272
AhHMA7.1   GSDTVLSQIISLVETAQMSKAPIQKFADYVASIFVPTVVALSLLTFLCWYTAGALEAYPKEWLPE....NGNHFVFALMF   274

                        *      340          *      360         *       380         *       400
AhHMA1.1   ALGLMVAASPCALAVA.PLAYATAISSCARKGILLKGGHVLDALASCHTIAFDKTGTLTTGGLLFKAIEPIYGHNVRKNE   332
AhHMA1.2   ALGLMVAASPCALAVA.PLAYATAISSCARKGILLKGGHVLDALASCHTIAFDKTGTLTTGGLLFKAIEPIYGHNVRKNE   332
AhHMA3.1   ALVVLVSACPCALILSTPVATFCAYTKAATSGLLIKGGDYLETLAKIKVMAFDKTGTITKGEFVVTNFVSLS........   300
AhHMA3.2   ALVVLVSACPCALILSTPVATFCAYTKAATSGLLIKGGDYLETLAKIKVMAFDKTGTITKGEFVVTNFVSLS........   300
AhHMA8.1   AVDVLVVSCPCALGLATPTAILVGTSLGARKGLLIRGGDVLERLASINYIALDKTGTLTKGKPVVSLVSSI.........   346
AhHMA8.2   AVDVLVVSCPCALGLATPTAILVGTSLGARKGLLIRGGDVLERLASINYIALDKTGTLTKGKPVVSSVSSI.........   323
AhHMA6.2   ACSVLVIACPCALGLATPTAVLVGTSLGATRGLLLRGGNILEKFAMVDTVVFDKTGTLTVGRPVVTKV...........   327
AhHMA6.1   ACSVLVIACPCALGLATPTAVLVGTSLGATRGLLLRGGNILEKFAMVDTVVFDKTGTLTVGRPVVTKV...........   362
AhHMA5.4   GISVMVIACPCALGLATPTAVMVGTGVGASQGVLIKGGQALESAHKVNCIVFDKTGTLTVGKPVVVNTKLFT........   348
AhHMA5.8   GISVMVIACPCALGLATPTAVMVGTGVGASQGVLIKGGQALESAHKVNCIVFDKTGTLTVGKPVVVNTKLFT........   348
AhHMA5.1   GISVMVIACPCALGLATPTAVMVGTGVGASQGVLIKGGQALESAHKVNCIVFDKTGTLTIGKPVVVNTKLLT........   348
AhHMA5.5   GISVMVIACPCALGLATPTAVMVGTGVGASQGVLIKGGQALESAHKVNCIVFDKTGTLTIGKPVVVNTKLLT........   348
AhHMA5.6   GISVMVIACPCALGLATPTAVMVGTGVGATQGVLIKGGQALESAHKVNCIVFDKTGTLTIGKPVIVTTKLFK........   342
AhHMA5.2   GISVMVIACPCALGLATPTAVMVGTGVGATQGVLIKGGQALESAHKVNCIVFDKTGTLTIGKPVIVTTKLFK........   342
AhHMA5.7   AISVLVVACPCALGLATPTAVMVASGMGASQGVLIKGGDALEKAHKVKVVVFDKTGTLTVGEPVVVSAVLFS........   348
AhHMA5.3   AISVLVVACPCALGLATPTAVMVASGMGASQGVLIKGGDALEKAHKVKVVVFDKTGTLTVGKPVVVSAVLFS........   348
AhHMA7.2   SIAVVVIACPCALGLATPTAVMVATGVGANNGVLIKGGDALERAQMVKYVIFDKTGTLTQGKASVTTAKTFT........   346
AhHMA7.3   SIAVVVIACPCALGLATPTAVMVATGVGANNGVLIKGGDALERAQMVKYVIFDKTGTLTQGKASVTTAKTFT........   346
AhHMA7.5   SIAVVVIACPCALGLATPTAVMVATGVGANNGVLIKGGDALERAQMVKYVIFDKTGTLTQGKASVTTAKTFT........   346
AhHMA7.4   SISVVVIACPCALGLATPTAVMVATGVGANNGVLIKGGDALERAQMVKYVIFDKTGTLTQGKATVTTVKVFT........   344
AhHMA7.1   SISVVVIACPCALGLATPTAVMVATGVGANNGVLIKGGDALERAQMVKYVIFDKTGTLTQGKATVTTVKVFT........   346

                        *      420          *      440         *       460         *       480
AhHMA1.1   SNISSCCIPTCEKEALAVAAAME...........KGTTHPIGRAVVDHSEGKD.......LP..............SVSV   380
AhHMA1.2   SNISSCCIPTCEKEALAVAAAME...........KGTTHPIGRAVVDHSEGKD.......LP..............SVSV   380
AhHMA3.1   .......DEIDLNTLLYWVSSIE...........SKSSHPLAAAIVDYGRSLS..IEPN.................PENV   343
AhHMA3.2   .......DDIDLNTLLYWVSSIE...........SKSSHPLAAAIVDYGRSLS..IEPN.................PENV   343
AhHMA8.1   ........NYGESEILQIAAAVE...........KTASHPIAKAIINKAESLE.......LV..............LPVT   386
AhHMA8.2   ........NYGESEILQIAAAVE...........KTASHPIAKAIINKAESLE.......LV..............LPVT   363
AhHMA6.2   .................IAPSCE...........QNANSSQAE.....................................   342
AhHMA6.1   .................IAPSCE...........QNANSSQAE.....................................   377
AhHMA5.4   ........NMVLRDFYELVAAAE...........VNSEHPLAKAVVEYAKKFK..DEEN.PS..............WLEA   392
AhHMA5.8   ........NMALRDFYELVAAAE...........ENSEHPLAKAVVEYAKKFK..DEEN.PS..............WLEA   392
AhHMA5.1   ........NMVLQEFYELVAAAE...........VNSEHPLAKAIVEYAKKLR..DEEN.PA..............WPEA   392
AhHMA5.5   ........NMVLQEFYELVAAAE...........VNSEHPLAKAIVEYAKKLR..DEEN.PV..............WPEA   392
AhHMA5.6   ........NMSVQDFYEL................ANSEHPIARAIVEHAKKIITEDEQN.HT..............WPEV   383
AhHMA5.2   ........NMSVQDFYELVAAAEASLLLLLNTTLVNSEHPIARAIVEHAKKIITEDEQN.HT..............WPEV   399
AhHMA5.7   ........EFTMEEFYDMTIAVE...........SSSEHPLAKAVVKHAKKLRKKFGSNDEE..............VPDV   395
AhHMA5.3   ........DFTMEEFYDMTIAVE...........SSSEHPLAKAVVKHAKKLRKKFGSNDEE..............VPDV   395
AhHMA7.2   ........GMQRGEFLRLVASAE...........ASSEHPLAQAIVEYARHFHFFDDSS.ASDGTQNGAKELKSGWLFDV   406
AhHMA7.3   ........GMQRGEFLRLVASAE...........ASSEHPLAQAIVEYARHFHFFDDSS.ASDGTQNGAKELKSGWLFDV   406
AhHMA7.5   ........GMQRGEFLRLVASAE...........ASSEHPLAQAIVEYARHFHFFDDSS.ASNGAQNGAKELKSGWLFDV   406
AhHMA7.4   ........GMERGEFLTLVASAE...........ASSEHPLGKAILQYARHFHFFDESS.TTNGTLNDAKELNSGWLHDV   404
AhHMA7.1   ........GMERGEFLTLVASAE...........ASSEHPLGKAILQYARHFHFFDESS.TTNGTLNDAKELSSGWLHDV   406

                        *      500          *      520         *       540         *       560
AhHMA1.1   ESFEYFPGRGLIATVNSIESGTGSAKLLKA....SLGSVDFITSFCHSEEESEKIKEAVNTSSYGSEFVHAALSVNKKVT   456
AhHMA1.2   ESFEYFPGRGLIATVNSIESGTGSAKLLKA....SLGSVDFITSFCHSEEESEKIKEAVNTSSYGSEFVHAALSVNKKVT   456
AhHMA3.1   TEFENFPGEGIYGKIEERLVYVGNKKIA...................TRAGSETVPAMRGQI..GKTTGYIYCG.ATPVG   401
AhHMA3.2   TEFENFPGEGIYGKIEERLVYVGNKKIA...................TRAGSEIVPAMHGQI..GKTTGYIYCG.ATPVG   401
AhHMA8.1   QGQLVEPGFGTLAEIDGRLVAVGSLEWVHDRFQIKMNPSDLVN....LEHTLMNHSSETSSNY.SKTVVYVGREGEGIIG   461
AhHMA8.2   QGQLVEPGFGTLAEIDGRLVAVGSLEWVHDRFQIKMNPSDLLN....LEHTLMNHSSETSSNY.SKTVVYVGREEEGIIG   438
AhHMA6.2   ..................................ENTWSDVEV....LRHGVSSKIHQELEDFKNQSVVYVGVN.DTLAG   383
AhHMA6.1   ..................................ENTWSDVEV....LRHGVSSKIHQELEDFKNQSVVYVGVN.DTLAG   418
AhHMA5.4   QDFVSITGHGVKATVRNQEIIVGNKSLMT.........SHSIA....VPSDAEDILAEAEAM..AQTGILVSIN.REVIG   456
AhHMA5.8   QDFVSITGHGVKATVRNQEIIVGNKSLMT.........SHSIA....VPSDAEDILAEAEAM..AQTGILVSIN.REVIG   456
AhHMA5.1   RDFVSITGHGVKATVRNKEVIVGNKNLMV.........EHNVV....VPADAEDILAEAEEM..AQTGILVSIS.GEVVG   456
AhHMA5.5   RDFVSITGHGVKATVRNKEVIVGNKNLMV.........EHNVV....VPADAEDILAEAEEM..AQTGILVSIS.GEVVG   456
AhHMA5.6   RDFVSISGHGVKAIVQNKEILAGNKKLMM.........DHNIA....ISVDAEEVLAEAERL..AQTGILVSLD.GEIAG   447
AhHMA5.2   RDFVSISGHGVKAIVQNKEILAGNKKLMM.........DHNIA....ISVDAEEVLAEAERL..AQTGILVSLD.GEIAG   463
AhHMA5.7   KDFEVQVGAGVCGKVGERTVLVGNKRLMH.........ACNVP....LSSEVERYMSENEIL..ARTCVLVSID.GKIAG   459
AhHMA5.3   KDFEVQVGAGVSGKVGERTVLVGNKRLMH.........ACNVP....LSSEVERYMSENEIL..ARTCVLVSID.GKIAG   459
AhHMA7.2   SDFIALPGRGVQCLIDGKPILVGNRKLMA.........ESGID....ISDEVENFVVELEES..ARTGVLVAYD.DIIIG   470
AhHMA7.3   SDFIALPGRGVQCLIDGKPILVGNRKLMA.........ESGID....ISDEVENFVVELEES..ARTGVLVAYD.DIIIG   470
AhHMA7.5   SDFIALPGKGVQCLIDGKPILVGNRKLMA.........ESGID....ISDEVENFVVELEES..ARTGVLVAYD.GIVIG   470
AhHMA7.4   SDFSALPGRGVQCFIDGKRILVGNRKLLS.........ENGID....ISTEVENFVVELEES..AKTGILVAYD.DVLTG   468
AhHMA7.1   SDFSALPGRGVQCFIDGKRILVGNRKLLS.........ENGID....ISTEVENFVVELEES..AKTGILVAYD.DVLTG   470
```

```
                          *        580         *        600         *        620         *        640
AhHMA1.1   LIHLEDRPRPGVFNVIQELQNEAMLRVIMLTGDHECSARRVANAVGISE...............................   505
AhHMA1.2   LIHLEDRPRPGVFNVIQELQNEAMLRVIMLTGDHECSARRVATAVGISE...............................   505
AhHMA3.1   FFSLSDACRTGAPEAIAQLKSLG.IKTAMLTGDSQGAAMQAQEQLGNALEL.............................   451
AhHMA3.2   FFSLSDACRTGVPEAIAQLKSLG.IKTAMLTGDSQGAAMQAQEQLGNALEL.............................   451
AhHMA8.1   SITISDVVREDAQSTVARLKQKG.IEMVLLSGDREEAVATIAQTVGIGSDF.............................   511
AhHMA8.2   SITISDVVREDAQSTVARLKQKG.IEMVLLSGDREEAVATVAQTVGIGSDF.............................   488
AhHMA6.2   LIYFEDEIREDAKHVVDQLSKQG.IGVYMLSGDKRNAAEHVASLVGISKEK.............................   433
AhHMA6.1   LIYFEDEIREDAKQVVDQLSKQG.IGVYMLSGDKRNAAEHVASLVGISKEKKLLHLHCKKCGATNFDDVDDPKPKHALCV   497
AhHMA5.4   VLAISDPLKPGAEEVISILKSVK.IRSIMVTGDNWGTANSIAREVGIET...............................   504
AhHMA5.8   VLAISDPLKPGAEEVISILKSVK.IRSIMVTGDNWGTANSIAREVGIET...............................   504
AhHMA5.1   VLAISDPLKPSAQEVISILRSVK.IRSIMVTGDNWGTANSIAKEVGIET...............................   504
AhHMA5.5   VLAISDPLKPSAQEVISILKSVN.IRSIMVTGDNWGTANSIAKEVGIET...............................   504
AhHMA5.6   VLAVSDPLKPDAKEVISILKSVK.IKSIMVTGDNWGTANSIARQAGIET...............................   495
AhHMA5.2   VLAVSDPLKPDAKEVISILKSVK.IKSIMVTGDNWGTANSIARQAGIET...............................   511
AhHMA5.7   AYSVTDPLKPEAKRVISFLHTVG.ISSIIATGDNLATATAIANEVGIDE...............................   507
AhHMA5.3   AYSVTDPLKPEAKRVISFLHSVG.ISSIIATGDNLATATAIANEVGIDE...............................   507
AhHMA7.2   VLGISDPLKREASVVIEGLQKVG.VRPVMVTGDNRRTARAVAKEVGIQD...............................   518
AhHMA7.3   VLGISDPLKREASVVIEGLQKVG.VRPVMVTGDNRRTARAVAKEVGIQD...............................   518
AhHMA7.5   VLGISDPLKREASVVIEGLQKVG.VRPVMVTGDNRRTARAVAKEVGIQD...............................   518
AhHMA7.4   VLGVADPLKREASVVIDGLHKVG.VIPVMVTGDNWRTARAVAKELGIQD...............................   516
AhHMA7.1   VLGVADPLKREASVVIDGLHKVG.VIPVMVTGDNWRTARAVAKELGIQD...............................   518

                          *        660         *        680         *        700         *        720
AhHMA1.1   ..............FHCNLKPEDKLSHVKDTS.......................................RDT   526
AhHMA1.2   ..............FHCNLKPEDKLSHVKDTS.......................................RDT   526
AhHMA3.1   ..............VHAELIPEDKLKIITEFK........................................KE   471
AhHMA3.2   ..............VHAELIPEDKLKIITEFK........................................KE   471
AhHMA8.1   ..............MKASLSPQQKSKFISSLK........................................AS   531
AhHMA8.2   ..............MKASLSPQQKSKFISSLK........................................AS   508
AhHMA6.2   ..............VLSGVKPDGKKKFINELQ........................................KD   453
AhHMA6.1   FDCCLLTAIHSHLVVLSGVKPDGKKKFINELQ........................................KD   531
AhHMA5.4   ..............VIAEAKPEQKAEKVKEFQ........................................AS   524
AhHMA5.8   ..............VIAEAKPEQKAEKVKEFQ........................................AS   524
AhHMA5.1   ..............VIAEAKPEQKAEKVKDLQ........................................AS   524
AhHMA5.5   ..............VNAEAKPEQKAEKVKDLQVHCFGRKKFSIHLHSLIKVLFQKDIDIIVVSKHIKWTFIYFSFFKKAS   570
AhHMA5.6   ..............VMAEAQPHTKATQVKDLQ........................................NS   515
AhHMA5.2   ..............VMAEAQPHTKATQVKDLQ........................................NS   531
AhHMA5.7   ..............MKGMT.........................................................   512
AhHMA5.3   ..............VFAETIPLGKADKVKELQ..........................................   525
AhHMA7.2   ..............VRAEVMPAGKADVVQSFQ........................................KD   538
AhHMA7.3   ..............VRAEVMPAGKADVVQSFQ........................................KD   538
AhHMA7.5   ..............VRAEVMPAGKADVVQSFQ........................................KD   538
AhHMA7.4   ..............VRAEVMPAGKADVVRSFQ........................................KD   536
AhHMA7.1   ..............VRAEVMPAGKADVVRSFQ........................................KD   538

                          *        740         *        760         *        780         *        800
AhHMA1.1   GGGLIMVGEGINDAPALAAATVGIVLAH.RASATAIAVADVLLLRENISAVPFCIAKSRQTTTLIKQNVALALFCITMAS   605
AhHMA1.2   GGGLIMVGEGINDAPALAAATVGIVLAH.RASATAIAVADVLLLRENISAVPFCIAKSRQTTTLIKQNVALALFCITMAS   605
AhHMA3.1   GP.TAMVGDGLNDAPALATADIGISMGI.SGSALATETGNIILMSNDLRKIPEVIKLAKKSHRKVIQNIFLSV.ITKAAI   548
AhHMA3.2   GP.TAMVGDGLNDAPALATADIGISMGI.SGSALASETGNIILMSNDLRKIPEVIKLAKKSHRKVIQNIFLSV.ITKAAI   548
AhHMA8.1   GHHVAMVGDGINDAPSLAVADVGIALQNEAQENAASDAASIILLGNKISHVVDALDLAQATMGKVYQNLSWAV.AYNVVA   610
AhHMA8.2   GHHVAMVGDGINDAPSLAVADVGIALQNEAQENAASDAASIILLGNKISHVVDALDLAQATMGKVYQNLSWAV.AYNVVA   587
AhHMA6.2   QKIVAMVGDGINDAAALASSHIGIALG..GGVGAASEVSSIVLMRNQLSQLVDALELSRLTMNTVKQNLWWAF.IYNIVG   530
AhHMA6.1   QKIVAMVGDGINDAAALASSHIGIALG..GGVGAASEVSSIVLMRNQLSQLVDALELSRLTMNTVKQNLWWAF.IYNIVG   608
AhHMA5.4   GKTVAMVGDGINDSPALVAADVGMAIG..AGTDIAIEAADIVLMKSNLEDVITAIDLSRKTFSRIRLNYFWAL.GYNLLG   601
AhHMA5.8   GHTVAMVGDGINDSPALVAADVGMAIG..AGTDIAIEAADIVLMKSNLEDVITAIDLSRKTFSRIRLNYFWAL.GYNLLG   601
AhHMA5.1   GCIVAMVGDGINDSPALVAADVGVAIG..AGTDIAIEAADIVLMKSNLEDVITAIDLSRKTFWRIRLNYIWAL.GYNLLG   601
AhHMA5.5   GCIVAMVGDGINDSPALVAADVGVAIG..AGTDIAIEAADIVLMKSNLEDVITAIDLSRKTFWRIRLNYIWAL.GYNLLG   647
AhHMA5.6   GYTVAMVGDGINDSPALVSADVGMAIG..AGTDIAIEAADIVLMRSNLEDIVIAIDLAKKTFNRIRLNYIWAL.GYNLLS   592
AhHMA5.2   GYTVAMVGDGINDSPALVSADVGMAIG..AGTDIAIEAADIVLMRSNLEDIVIAIDLAKKTFNRIRLNYIWAL.GYNLLS   608
AhHMA5.7   ...VAMVGDGINDSPALVAADVGMAIG..AGTDVAIEAADIVLVKNSLEDVITAIDLSRKTMLRIRLNYIWAL.GYNVLG   586
AhHMA5.3   ............................G.....................................................   526
AhHMA7.2   GSIVAMVGDGINDSPALAAADVGMAIG..AGTDIAIEAADYVLMRNNLEDVITAIDLSRKTFSRIRLNYVFAI.VYNVVA   615
AhHMA7.3   GSIVAMVGDGINDSPALAAADVGMAIG..AGTDIAIEAADYVLMRNNLEDVITAIDLSRKTFSRIRLNYVFAI.VYNVVA   615
AhHMA7.5   GSIVAMVGDGINDSPALAAADVGMAIG..AGTDIAIEAADYVLMRNNLEDVITAIDLSRKTFSRIRLNYVFAM.VYNVVA   615
AhHMA7.4   GSIVAMVGDGINDSPALAAADVGLAIG..AGTDIAIEAADYVLMRDNLEDVITAIDLSRKTFSRIRLNYVFAM.AYNVIA   613
AhHMA7.1   GSVVAMVGDGINDSPALAAADVGMAIG..AGTDIAIEAADYVLMRYNLEDVITAIDLSRKTFSRIRLNYVFAM.AYNVIA   615
```

Figure S2. Complete multiple sequence alignment of the 21 AhHMA proteins using MAFFT (v. 7) with the default settings. Identical residues are highlighted in black and homologous residues in gray, while the typical conserved amino acid residues such as TGE, CPC/SPC, DKTGTLT, HP, PxxK, and GDGxNDxP are highlighted in red lines.

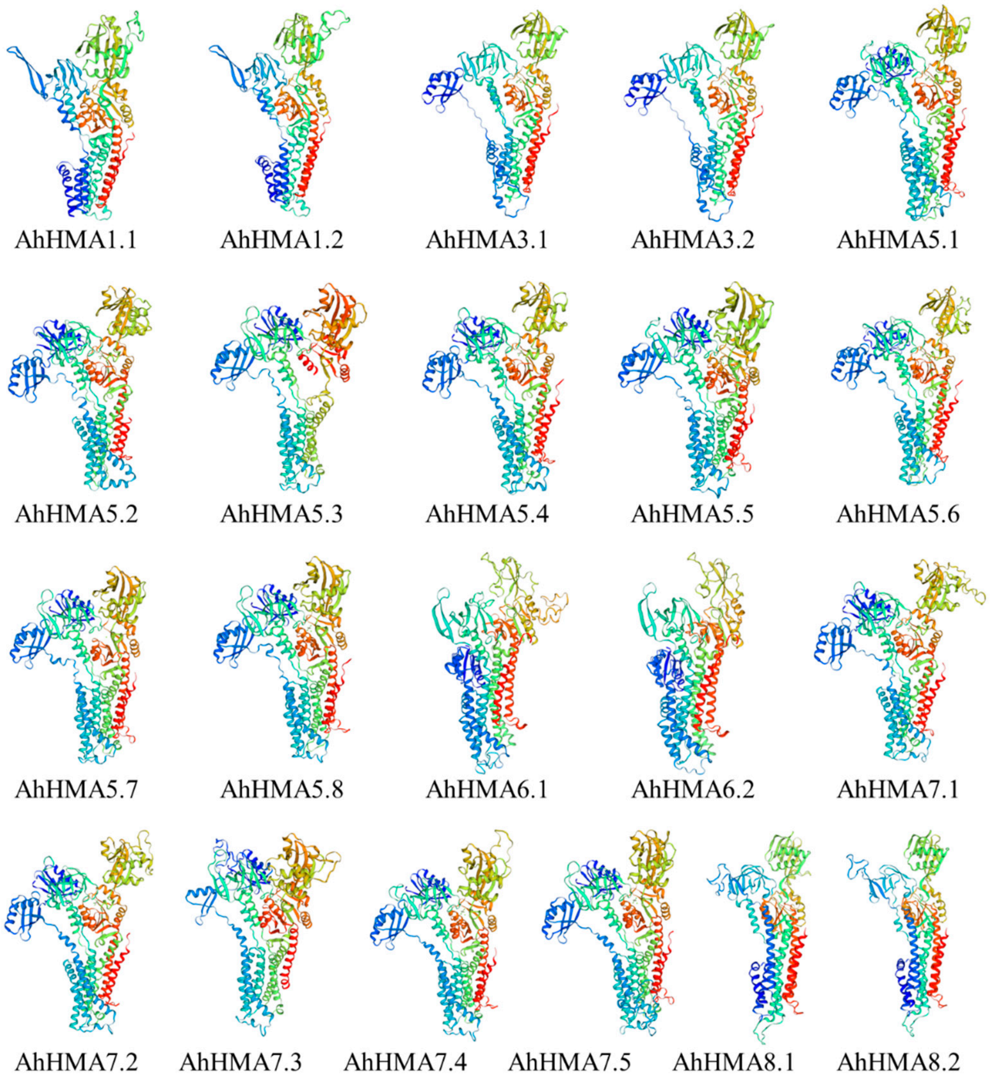

Figure S3. Predicted 3D structure of peanut AhHMA proteins by SwissModel. Models were visualized by rainbow color from N to C terminus.
